# CD19-Targeted Chimeric Antigen Receptor T-cell Therapy for Concomitant Diffuse Large B-cell Lymphoma and Multiple Myeloma

**DOI:** 10.7759/cureus.44542

**Published:** 2023-09-01

**Authors:** Tyler D'Ovidio, Kathryn Ciccolini, Matko Kalac, Keren Osman, Amir Steinberg

**Affiliations:** 1 Division of Hematology and Medical Oncology, Icahn School of Medicine at Mount Sinai, New York, USA

**Keywords:** car t-cell therapy, diffuse large b-cell lymphoma, non-hodgkin’s lymphoma, concomitant malignancies, multiple myeloma

## Abstract

Multiple myeloma (MM) and diffuse large B-cell lymphoma (DLBCL) comprise a large fraction of hematologic malignancies diagnosed each year. However, the co-occurrence of these conditions in the same patient is rare. CD19- and B-cell maturation antigen-targeted chimeric antigen receptor (CAR) T-cell therapies have been approved in recent years with promising responses. Here, we present a patient who presented following a bone marrow biopsy that revealed MM with 20% lambda-restricted plasma cells with no evidence of lymphoma involvement in the marrow. A subsequent lymph node biopsy of a right thigh mass was done and revealed DLBCL. The patient received CD19-targeted CAR T-cell therapy and has no detectable MM or DLBCL. To our knowledge, this is the first case report in the literature describing a patient with concomitant MM and DLBCL who received CD19-targeted CAR T-cell therapy.

## Introduction

Multiple myeloma (MM) is a neoplasm of plasma cells in the bone marrow and comprises approximately 17% of annual hematologic malignancies. Non-Hodgkin lymphoma represents a diverse group of malignancies derived from lymphocytes and represents approximately 43% of annual hematologic malignancies [1]. Within this group, diffuse large B-cell lymphoma (DLBCL) is the most common subtype [2,3]. The co-occurrence of these malignancies is rare, with approximately one in 804 patients with MM developing B-cell lymphoma and one in 700 patients with B-cell lymphoma developing MM [4].

In 2017, The U.S. Food and Drug Administration (FDA) approved two CD19-targeted chimeric antigen receptor (CAR) T-cell therapies. The first to be approved was Kymriah (tisagenlecleucel) for B-cell acute lymphoblastic leukemia patients up to age 25 [5]. The second CAR T-cell therapy to be approved was Yescarta (axicabtagene ciloleucel) to treat adult patients with DLBCL who have not responded to or have relapsed after at least two other kinds of treatment [6]. For DLBCL patients who are ineligible or not good candidates for autologous hematopoietic stem cell transplantation (HSCT), CAR T is a reasonable option with promising long-term efficacy. Recent trials have demonstrated the utility of B-cell maturation antigen (BCMA)-targeted CAR T-cell therapy for myeloma, and in March 2021, the FDA approved Abecma (idecabtagene vicleucel) for myeloma patients who have been exposed to four prior lines of therapy [7-9]. We present a case of concurrent multiply relapsed/refractory (r/r) DLBCL and non-r/r MM treated exclusively with CD19-targeted CAR T-cell therapy.

This article was previously accepted as an abstract without presentation at the 63rd ASH Annual Meeting and Exposition from December 11, 2021, to December 14, 2021. This is a follow-up full case report to that abstract.

## Case presentation

A 77-year-old female with a previous medical history of hypertension, osteoporosis on vitamin D and calcium supplementation, left femoral deep venous thrombosis, and chronic kidney disease was admitted to another hospital with a chief complaint of one week of constipation and incidental labs demonstrating hypercalcemia (serum calcium of 16.8 mg/dL) without discrete lytic bone lesions on positron emission tomography/computed tomography (PET/CT) and acute kidney injury (creatinine of 2.99 mg/dL). She underwent a bone marrow biopsy due to high suspicion for malignancy. There were 20% lambda-restricted plasma cells without morphologic evidence of lymphoma. Immunohistochemistry results demonstrated the following immunophenotype: CD138+, MUM-1+, CD56-, and CD117 (KIT)-. Unfortunately, because the initial bone marrow biopsy was performed at another hospital, fluorescence in situ hybridization results were not obtainable. She also had concomitant anemia with a hemoglobin of 9.6 g/dL best attributed to plasma cell dyscrasia, thus fulfilling the International Myeloma Working Group (IMWG) criteria [10]. Serum beta-2-microglobulin was 6.8 mg/L and lactate dehydrogenase was 877 U/L. She demonstrated two M proteins at the time of diagnosis, each being 0.40 g/dL. Interestingly, these monoclonal proteins were IgG-kappa and an IgM protein. She fulfilled the revised International Staging System for Stage III myeloma disease [11]. She received a single cycle of bortezomib, cyclophosphamide, and dexamethasone before transfer to our institution.

Upon receiving the patient, we performed a right inguinal lymph node biopsy of an existing (first noted in 2014, the patient was lost to follow-up at this time) right thigh mass which revealed atypical cells with the following immunophenotype: CD20+, PAX5+ (weak), MUM1+, BCL2+, CD10-, CD5-, BCL6-, BCL1-, and cMYC-. The findings were consistent with non-GC DLBCL (Revised International Prognostic Index 4, CNS-IPI 4, Stage IV). The patient underwent a PET/CT scan, revealing avid uptake in the biopsied right upper thigh soft tissue mass, as well as in the right tibia and soft tissue of the right lower leg. Weighing the kinetics of the patient’s diseases and the role that lymphoma chemotherapy can play in killing myeloma cells, chemotherapy with cyclophosphamide, doxorubicin, vincristine, prednisone, and rituximab (R-CHOP) was initiated and continued for four cycles resulting in a mixed response. Following R-CHOP, her serum M protein level was 0.13 g/dL. However, urine studies were not performed, and the myeloma response at this time was classified as a stable disease per the IMWG criteria [10]. The following month, the patient was hospitalized with hypercalcemia. A second right inguinal lymph node biopsy revealed immunohistochemistry consistent with refractory DLBCL (positive for CD79a, CD20 (weak), MUM1, BCL2, BCL6, MIB1 Ki67 60-70% proliferative fraction, CD30 (1-2%), while negative for CD10, CD3, CD5, BCL1, and MYC). The patient received rituximab, ifosfamide, carboplatin, and etoposide (RICE) given as second-line therapy. After the RICE therapy, the patient’s DLBCL was classified as a stable disease per Lugano criteria [12]. Radiation therapy was subsequently performed due to the unsatisfactory response of her DLBCL to RICE and as a palliative measure to reduce the patient’s discomfort and pain associated with her disease. Pertinently, the monoclonal protein did resolve after the patient’s second cycle of RICE; however, urine studies were not performed, and her myeloma remained classified as a stable disease per the IMWG criteria [10]. A repeat biopsy of the right thigh mass was then obtained and indicated a CD19+ specimen on review, and the patient was referred to CD19-targeted CAR T-cell therapy. Polatuzumab vedotin was initiated as a bridging therapy for one dose while the patient’s CAR T-cell product was manufactured. This was followed by a cyclophosphamide and fludarabine lymphodepletion regimen and CAR T-cell infusion. The day following CAR T-cell infusion, the patient developed grade 1 cytokine release syndrome [13], which was managed with tocilizumab, acetaminophen, and empiric cefepime. The patient also developed grade 2 immune effector cell-associated neurotoxicity syndrome five days after CAR T-cell infusion [14], managed with dexamethasone, anakinra, and prophylactic levetiracetam. Lastly, the patient developed neutropenia over the week following CAR T-cell infusion (absolute neutrophil count 2,300/µL) which was treated with granulocyte colony-stimulating factor. The patient was discharged from the hospital 13 days after CAR T-cell infusion.

Per Lugano criteria, the patient achieved a successful partial response for lymphoma through her 90-day evaluation (Deauville 3) [12]. Her serum immunofixation was positive as late as 16 days after CAR T-cell infusion, and subsequent testing done 32 days after CAR T-cell infusion no longer detected any abnormality. Three months after CAR T-cell therapy, the M protein level was 0.00 g/dL and she had no detectable monoclonal protein on serum immunofixation or electrophoresis testing. The patient’s response at this point in the treatment, according to the IMWG criteria, her myeloma would technically be classified as a stable disease as urine M protein testing was not conducted [10]. Flow cytometry peripheral blood smear was negative for plasma cells by CD38 and CD138 labeling seven months after CAR T-cell infusion, and the patient remained in partial response. At nine months after CAR T-cell therapy, both her serum and urine immunofixation and electrophoresis were negative. At 28 months after CAR T-cell infusion, a repeat bone marrow biopsy demonstrated less than 5% plasma cells without kappa or lambda restriction, suggesting nonclonal plasma cells and a continued excellent response of her myeloma. At present, the patient is 34 months status post-CAR T-cell therapy and remains in partial response for DLBCL per Lugano criteria [12].

## Discussion

CD19-targeted CAR T-cell therapy has become an established and accepted standard of care for multiply r/r DLBCL when it is available [15]. However, the role of HSCT in r/r DLBCL is as the standard of care after second-line salvage [16]. Our patient, however, failed to adequately respond to salvage chemotherapy. Though there is data supporting the use of HSCT in chemorefractory patients, it is not as successful as in chemosensitive disease [17]. Although several trials have investigated BCMA-targeted CAR T-cell therapy in r/r MM and have reported high response rates [18], there is a lack of data supporting these immunologic therapies in non-r/r MM; however, studies are ongoing [19]. Data regarding the effect of CD19-targeted CAR T-cell on MM is limited as secondary malignancies usually exclude subjects from enrollment; however, CD19-mediated responses have been reported in vitro, and CD19-targeted clinical trials are underway in r/r MM (NCT02135406) [20-22]. Furthermore, Li et al. provided a report of a single patient presenting with existing mucosa-assisted lymphoid tissue lymphoma and subsequent development of MM. In this case, administration of both CD19- and BCMA-targeted CAR T-cell therapies resulted in effective disease control [23].

Identifying the malignancy underlying our patient’s presentation was essential in initiating appropriate management. While pathology confirmed DLBCL, the tumor was also characterized to be CD5-, CD10-, and BCL6-. The patient was noted to have had a thigh mass since 2014, and it may be reasonable to speculate that what began as a marginal-zone lymphoma transformed over time into DLBCL [24]. The morphology of the lymphoma was not consistent with plasmablastic differentiation or lymphoplasmablastic lymphoma. Additionally, our patient was HIV-negative and the lymphoma’s immunophenotype was CD20+. While not ruling out plasmablastic lymphoma, these factors do decrease the likelihood substantially [25].

Based on our evaluation of the patient and the emerging data regarding the effectiveness of CAR T-cell therapy, our team elected to treat the more aggressive DLBCL and pursue CD19-targeted CAR T-cell therapy. One point of critique is the discrepancy between the monoclonal protein detected in the blood, which differed from the lambda-restricted plasma cell population observed in the initial bone marrow biopsy. While a repeat bone marrow biopsy was initially deferred given the lack of detectable myeloma based on laboratory tests and imaging (no bone lesions on PET/CT), the patient’s recent biopsy confirmed reduction in marrow plasma cells, and laboratory tests continue to demonstrate resolution of serum and urine M protein. An additional critique is the lack of urine testing to have an official IMWG criteria response immediately following each of the patient’s therapies as it is difficult to highlight the temporality of the myeloma’s remission. Lastly, it is essential to acknowledge that our findings are based on a single patient case and should be interpreted with prudence.

## Conclusions

To our knowledge, this is the first report of a patient with concurrent MM and DLBCL treated with CD19-targeted CAR T-cell therapy. This patient achieved a successful partial response and will continue to be monitored for disease status. Our patient case shows that directing therapy toward the more aggressive malignancy, typically the lymphoma with CAR T-cell therapy, can target the myeloma as well. This has significant implications for informing treatment decisions in future patients presenting with concomitant malignancies. Furthermore, emerging data from studies investigating the effect of CD19-targeted CAR T-cell therapy on myeloma will be of great clinical interest.

## References

[1] Siegel RL, Miller KD, Jemal A (2020). Cancer statistics, 2020. CA Cancer J Clin.

[2] Swerdlow SH, Campo E, Pileri SA (2016). The 2016 revision of the World Health Organization classification of lymphoid neoplasms. Blood.

[3] Morton LM, Wang SS, Devesa SS, Hartge P, Weisenburger DD, Linet MS (2006). Lymphoma incidence patterns by WHO subtype in the United States, 1992-2001. Blood.

[4] Mahindra AK, Sohani AR, Toomey CE (2011). B cell lymphoma in association with multiple myeloma: analysis of the biologic relationship. Blood.

[5] Liu Y, Chen X, Han W, Zhang Y (2017). Tisagenlecleucel, an approved anti-CD19 chimeric antigen receptor T-cell therapy for the treatment of leukemia. Drugs Today (Barc).

[6] Locke FL, Ghobadi A, Jacobson CA (2019). Long-term safety and activity of axicabtagene ciloleucel in refractory large B-cell lymphoma (ZUMA-1): a single-arm, multicentre, phase 1-2 trial. Lancet Oncol.

[7] Madduri D, Usmani SZ, Jagannath S (2019). Results from CARTITUDE- 1: a phase 1b/2 study of JNJ-4528, a CAR-T cell therapy directed against B-cell maturation antigen (BCMA), in patients with relapsed and/or refractory multiple myeloma (R/R MM). Blood.

[8] Munshi NC, Anderson LD Jr, Shah N (2021). Idecabtagene vicleucel in relapsed and refractory multiple myeloma. N Engl J Med.

[9] Raje N, Berdeja J, Lin Y (2019). Anti-BCMA CAR T-cell therapy bb2121 in relapsed or refractory multiple myeloma. N Engl J Med.

[10] Rajkumar SV, Dimopoulos MA, Palumbo A (2014). International Myeloma Working Group updated criteria for the diagnosis of multiple myeloma. Lancet Oncol.

[11] Palumbo A, Avet-Loiseau H, Oliva S (2015). Revised International Staging System for multiple myeloma: a report from International Myeloma Working Group. J Clin Oncol.

[12] Cheson BD, Ansell S, Schwartz L (2016). Refinement of the Lugano Classification lymphoma response criteria in the era of immunomodulatory therapy. Blood.

[13] Lee DW, Gardner R, Porter DL (2014). Current concepts in the diagnosis and management of cytokine release syndrome. Blood.

[14] Lee DW, Santomasso BD, Locke FL (2019). ASTCT consensus grading for cytokine release syndrome and neurologic toxicity associated with immune effector cells. Biol Blood Marrow Transplant.

[15] Munshi PN, Ujjani C (2019). The acceleration of CAR-T therapy in non-Hodgkin lymphoma. Hematol Oncol.

[16] Philip T, Guglielmi C, Hagenbeek A (1995). Autologous bone marrow transplantation as compared with salvage chemotherapy in relapses of chemotherapy-sensitive non-Hodgkin's lymphoma. N Engl J Med.

[17] Vardhana SA, Sauter CS, Matasar MJ (2017). Outcomes of primary refractory diffuse large B-cell lymphoma (DLBCL) treated with salvage chemotherapy and intention to transplant in the rituximab era. Br J Haematol.

[18] Lin Q, Zhao J, Song Y, Liu D (2019). Recent updates on CAR T clinical trials for multiple myeloma. Mol Cancer.

[19] (2018). Pennsylvania Uo, Novartis: up-front CART-BCMA with or without huCART19 in high-risk multiple myeloma. https://ClinicalTrials.gov/show/NCT03549442.

[20] Feng D, Sun J (2020). Overview of anti-BCMA CAR-T immunotherapy for multiple myeloma and relapsed/refractory multiple myeloma. Scand J Immunol.

[21] Nerreter T, Letschert S, Götz R (2019). Super-resolution microscopy reveals ultra-low CD19 expression on myeloma cells that triggers elimination by CD19 CAR-T. Nat Commun.

[22] Pennsylvania Uo (2014). CART-19 for multiple myeloma. https://ClinicalTrials.gov/show/NCT02135406.

[23] Li T, Tan J, Chen L (2020). Case report: simultaneous occurrence of multiple myeloma and non-Hodgkin lymphoma treated by CAR T therapy. Medicine (Baltimore).

[24] Campo E, Nathwani BN, Pileri SA, Stein H, Jaffe ES, Muller-Hermelink HK (2017). Nodal marginal zone lymphoma. WHO Classification of Tumours of Haematopoietic and Lymphoid Tissues.

[25] Campo E, Nathwani BN, Pileri SA, Stein H, Jaffe ES, Muller-Hermelink HK (2017). Plasmablastic lymphoma. WHO Classification of Tumours of Haematopoietic and Lymphoid Tissues.

